# Epigenetic Age in Peripheral Blood Among Children, Adolescent, and Adult Survivors of Childhood Cancer

**DOI:** 10.1001/jamanetworkopen.2023.10325

**Published:** 2023-04-28

**Authors:** Noel-Marie Plonski, Cheng Chen, Qian Dong, Na Qin, Nan Song, Hemang M. Parikh, Kyla Shelton, Emily R. Finch, John Easton, Heather Mulder, Jinghui Zhang, Geoffrey Neale, Emily Walker, Hui Wang, Kevin Krull, Kirsten K. Ness, Melissa M. Hudson, Leslie L. Robison, Qian Li, AnnaLynn Williams, Zhaoming Wang

**Affiliations:** 1 Department of Epidemiology and Cancer Control, St Jude Children’s Research Hospital, Memphis, Tennessee; 2 School of Public Health, Shanghai Jiaotong University, Shanghai, China; 3 Department of Epidemiology, Center for Global Health, School of Public Health, Nanjing Medical University, Nanjing, Jiangsu, China; 4 College of Pharmacy, Chungbuk National University, Cheongju, Korea; 5 Health Informatics Institute, Morsani College of Medicine, University of South Florida, Tampa; 6 Department of Computational Biology, St Jude Children’s Research Hospital, Memphis, Tennessee; 7 Hartwell Center, St Jude Children’s Research Hospital, Memphis, Tennessee; 8 Department of Oncology, St Jude Children’s Research Hospital, Memphis, Tennessee; 9 Department of Biostatistics, St Jude Children’s Research Hospital, Memphis, Tennessee; 10 Division of Supportive Care in Cancer, Department of Surgery, University of Rochester Medical Center, Rochester, New York

## Abstract

**Importance:**

Certain cancer therapies are risk factors for epigenetic age acceleration (EAA) among survivors of childhood cancer, and EAA is associated with chronic health conditions (CHCs). However, small numbers of younger survivors (aged <20 years) previously evaluated have limited the ability to calculate EAA among this age group.

**Objective:**

To evaluate the change rate of epigenetic age (EA) and EAA in younger compared with older survivors and the possible association of EAA with early-onset obesity (aged <20 years), severity/burden of CHCs, and late mortality (>5 years from cancer diagnosis).

**Design, Setting, and Participants:**

Study participants were from the St Jude Lifetime Cohort, initiated in 2007 with ongoing follow-up. The present study was conducted from April 17, 2022, to March 23, 2023. Survivors in this cohort of European ancestry with DNA methylation data were included. Cross-sectional annual changes in EA and EAA were compared across 5 different chronologic age groups: age 0 to 9 (children), 10 to 19 (adolescents), 20 to 34 (younger adults), 35 to 49 (middle-aged adults), and greater than or equal to 50 (older adults) years. Logistic regression evaluated the association between EAA and early-onset obesity or severity/burden of CHCs. Cox proportional hazards regression assessed the association between EAA and late mortality.

**Main Outcomes and Measures:**

Early-onset obesity, severity/burden of CHCs (graded using the Common Terminology Criteria for Adverse Events (grade 1, mild; 2, moderate; 3, severe/disabling; 4, life-threatening) and were combined into high vs low severity/burden based on frequency and grade), and late mortality were the outcomes based on follow-up until April 2020. Expanded DNA methylation profiling increased the number of survivors younger than 20 years (n = 690). Epigenetic age was calculated primarily using the Levine clock, and EAA was derived from least squares regression of EA against chronologic age and was standardized to a *z* score (Levine EEA).

**Results:**

Among 2846 participants (median age, 30.3 [IQR, 9.3-41.5] years; 53% males), the cross-sectional annual change in EA_Levine was higher in children (1.63 years) and adolescents (1.14 years), and the adjusted least-squares mean of Levine EEA was lower in children (−0.22 years) and older adults (−1.70 years). Each 1-SD increase in Levine EEA was associated with increased risk of developing early-onset obesity (odds ratio [OR], 1.46; 95% CI, 1.19-1.78), high severity/burden of CHCs (OR, 1.13; 95% CI, 1.03-1.24), and late mortality (hazard ratio, 1.75; 95% CI, 1.35-2.26).

**Conclusions and Relevance:**

The findings of this study suggest that EAA measured in children and adolescent survivors of childhood cancer is associated with early-onset obesity, severity/burden of all CHCs, and late mortality. Evaluating EAA may help identify survivors of childhood cancer at increased risk for early-onset obesity, morbidity in general, and mortality.

## Key Points

**Question:**

Is the change rate of epigenetic age (EA) and EA acceleration different in younger (children and adolescents) survivors of childhood cancer vs adults?

**Findings:**

In this cross-sectional study of 2846 childhood cancer survivors, the change rate of EA and EA acceleration among younger survivors was different from the rate in adult survivors. Greater EA acceleration measured in younger survivors was associated with increased early-onset obesity, severity/burden of morbidity, and late mortality.

**Meaning:**

The findings of this study suggest that evaluating EA acceleration among younger survivors of childhood cancer may identify those at increased risk for early-onset obesity, morbidity burden in general, and late mortality, and inform an early entry point for antiaging intervention.

## Teaser

This cross-sectional study evaluates the occurrence of obesity, chronic health conditions, and mortality associated with epigenetic age acceleration in individuals who survived childhood cancer.

## Introduction

Mechanistic research such as cell reprogramming gave rise to an emerging view that epigenetic alteration is likely the prime mechanism involved in aging.^1^ In theory, epigenetic alterations are reversible, providing a promising opportunity for an antiaging intervention strategy called epigenetic rejuvenation.^2^ Since the inception of the epigenetic clock in 2013, there have been dozens of epigenetic clocks proposed in the field of aging biomarker research.^3^ The first generation of epigenetic clocks was based on models predicting chronologic age (CA), such as the Horvath clock,^4^ which comprises 353 CpGs and was developed using blood and other tissue samples from a wide age range of individuals, including children and adolescents, and the Hannum clock,^5^ which comprises 71 CpGs and was developed using whole blood samples only. The second generation was based on models predicting physiologic/biologic age, for example, the Levine clock,^6^ which comprises 513 CpGs and predicts composite phenotypic age (considering CA and other clinical biologic markers, such as glucose, C-reactive protein, albumin, and creatinine levels; lymphocyte percentage; mean cell volume; red cell distribution; alkaline phosphatase level; and white blood cell count), and GrimAge,^7^ which comprises 1030 CpGs and predicts mortality with DNA methylation (DNAm) surrogates for multiple biologic measurements as well as pack-years of smoking.

Survivors of childhood cancer, who number more than half a million in the US today,^8^ are at increased risk for high morbidity from chronic health conditions (CHCs) and for mortality rates typically seen among individuals decades older in the general population, suggesting an accelerated aging phenotype.^9,10^ However, there are limited studies applying molecular biomarkers to quantify the acceleration of aging in this population. A study including some of us reported that epigenetic age acceleration (EAA), a residual obtained from regressing epigenetic age (EA) on CA, is significantly higher in survivors than in individuals without a history of cancer and is associated with cancer treatment exposures, unfavorable health behaviors, and the presence of certain CHCs, such as obesity, hypertension, and myocardial infarction.^11^ However, a limited number of survivors (n = 141) had EAA measured when they were younger than 20 years, which prevented evaluation of these associations in younger survivors.

The St Jude Lifetime Cohort (SJLIFE) has recently expanded DNAm profiling data to cover the age range of children and adolescent survivors (aged <20 years). Using the new data, we examined how the cross-sectional annual changes in EA with respect to CA among children and adolescent survivors compared with adult survivors and whether the EAA differed by CA-defined groups. We also examined the association between EAA and early-onset obesity that occurs before the age of 20 years because it is one of the most common CHCs in childhood cancer survivors and because obesity is linked to multiple CHCs in this population.^12,13,14^ In addition, we evaluated associations between EAA and severity/burden of all CHCs or late mortality. Understanding contributions of EAA during childhood and adolescence to the development of morbidity and mortality later in life may help identify survivors most in need of antiaging interventions that promote epigenetic rejuvenation.

## Methods

### Study Population

The study participants were childhood cancer survivors from the SJLIFE study, initiated in 2007 with ongoing follow-up. The SJLIFE study was approved by the St Jude Institutional Review Board, and written informed consent was obtained from each study participant who provided blood samples for research use. The study design and clinical assessments of phenotypes have been previously described.^15,16,17^ Briefly, eligible participants included those who survived 5 years or more after diagnosis of a childhood cancer treated at St Jude Children’s Research Hospital between 1962 and 2012. A total of 2846 survivors of European ancestry (2138 from the previous reported study^11^ and 708 with newly generated DNAm data, as described in a more recent study^18^) were included in the current analysis, which was conducted from April 17, 2022, to March 23, 2023. Individuals of other racial ancestry were not considered because the total number was too small for age-stratified analysis. With the additional DNAm data, the number of survivors who had EA/EAA measured when they were younger than 20 years increased from 141 to 690. Participants were divided into 5 chronologic age groups (denoted as CA-defined groups): 0 to 9 (children), 10 to 19 (adolescents), 20 to 34 (younger adults), 35 to 49 (middle-aged adults), and greater than or equal to 50 (older adults) years. This study followed the Strengthening the Reporting of Observational Studies in Epidemiology (STROBE) reporting guideline.

Demographic information (race and ethnicity and sex) was collected from the questionnaires. Treatment exposure information was extracted from medical records using a structured protocol,^15^ including region-specific radiotherapy (RT), such as the brain, neck, chest, and abdomen or pelvis, and chemotherapeutic agents, including alkylating agents, anthracyclines, and epipodophyllotoxins, previously shown to be associated with EAA.^11^ The data are accessible through the St Jude Cloud.^19^

### Epigenetic Age and EAA

Along with EA_Levine (Levine clock) as the primary measurement, 3 other epigenetic clocks, including EA_Horvath (Horvath clock), EA_Hannum (Hannum clock), and EA_GrimAge (GrimAge), were estimated with the new DNAm age calculator.^20^ The EAA was calculated as residuals of a least squares regression model (epigenetic age vs age at DNA sampling) and was standardized to a *z* score. Additionally, delta age (ie, EA – CA), another method of determining EAA,^21^ was calculated as a comparison to using residuals from the least squares regression model.

### Health Outcomes

Severity/burden counts of CHCs were initially derived from 216 graded conditions using a modification of the National Cancer Institute Common Terminology Criteria for Adverse Events (CTCAE), v.4.03, and categorized as none/low for those with grade 1 conditions only, medium for those with 1 or more grade 2 or 1 grade 3 conditions, high for those with 2 or more grade 3 or 1 grade 4 or 1 grade 4 and 1 grade 3 conditions, and very high for those with 2 or more grade 4 or 2 or more grade 3 and 1 grade 4 conditions.^22^ In this study, none/low and medium categories were combined to represent a low severity/burden score, and high and very high categories were combined to represent a high severity/burden score. The CHCs were based on follow-up clinical assessments through April 30, 2020. Early-onset obesity was defined as CTCAE grade 2 or 3 obesity before age 20 years. Mortality data were obtained through a search of the National Death Index through December 31, 2016.

### Statistical Analysis

A linear regression of EA against CA, sex, treatment exposures, and CA-defined groups was performed, where the difference in the annual change of EA with respect to CA was tested across CA-defined groups by introducing an interaction (CA × CA-defined groups). Adjusted least squares mean (ALSM) and SE of EAA for each CA-defined group were calculated from a multivariable generalized linear regression of EAA against CA groups, sex, and treatment exposures.

Among children and adolescents, early-onset obesity (aged <20 years) was analyzed in a logistic regression model for assessing the association with EAA (measured when <20 years and before the age at assessment for obesity) adjusting for sex and treatment exposures. Logistic regression also estimated the association between severity/burden score of CHCs and EAA adjusting for sex, treatment exposures, attained age, with and without adjusting for CA groups. A multivariable Cox proportional hazards regression was performed to model the time from DNA sampling for EAA measurement to late mortality, adjusting for sex, age at DNA sampling, and treatment exposures. Few survivors (<2%) with missing cancer treatment information were excluded from the analysis. *P* value was based on a 2-sided test, and *P* < .05 was deemed as the level of statistical significance. All analyses were performed with R, version 3.6.1 (R Foundation for Statistical Computing).

## Results

### Study Population

Of the 2846 participants (Table 1), 53.0% were male and 47.0% were female. Primary diagnoses comprised leukemia (32.6%), lymphoma (18.0%), sarcoma (12.6%), central nervous system tumors (13.5%), embryonal tumors (13.7%), and other solid tumors (6.3%). Among the participants, 56.7% received anthracyclines, 56.7% received alkylating agents, and 34.1% received epipodophyllotoxins; 25.2% were exposed to brain RT, 20.2% to chest RT, 17.9% to abdomen RT, and 15.5% to pelvis RT. The median age at DNA sampling was 29.1 (IQR, 20.2-37.4) years, and at last follow-up was 30.3 (IQR, 9.3-41.5 years).

**Table 1.** Characteristics of the Study Population

Characteristic	No. (%)		
	All participants (N = 2846)	Chronologic age at blood draw, y	
		<20 (n = 690)	≥20 (n = 2156)
Sex			
Male	1509 (53.0)	358 (51.9)	1151 (53.4)
Female	1337 (47.0)	332 (48.1)	1005 (46.6)
Diagnosis			
Leukemia	927 (32.6)	204 (29.6)	727 (33.7)
Lymphoma	512 (18.0)	25 (3.6)	487 (22.6)
Sarcoma	358 (12.6)	67 (9.7)	291 (13.5)
CNS tumors	383 (13.5)	141 (20.4)	242 (11.2)
Embryonal^a^	391 (13.7)	114 (16.5)	277 (12.8).
Other	271 (9.5)	139 (20.1)	132 (6.1)
Treatment			
Anthracycline	1613 (56.7)	349 (50.6)	1264 (58.6)
Alkylating agent	1614 (56.7)	344 (49.9)	1270 (58.9)
Epipodophyllotoxin	971 (34.1)	218 (31.6)	753 (34.9)
Brain RT	718 (25.2)	93 (13.5)	625 (29.0)
Chest RT	574 (20.2)	60 (8.7)	514 (23.8)
Abdomen RT	509 (17.9)	75 (10.9)	434 (20.1)
Pelvis RT	440 (15.5)	60 (8.7)	380 (17.6)
CA-defined groups			
Children	178 (6.3)	178 (25.8)	NA
Adolescents	512 (18.0)	512 (74.2)	NA
Younger adults	1285 (45.2)	NA	1285 (59.6)
Middle-aged adults	749 (26.3)	NA	749 (34.7)
Older adults	122 (4.3)	NA	122 (5.7)
Age at DNA sampling, median (IQR), y	29.1 (20.2-37.4)	12.9 (9.9-16.1)	32.4 (27.2-40.1)
Age at follow-up, median (IQR), y	30.3 (9.3-41.5)	9.8 (7.3-17.6)	34.1 (10.0-42.8)

Abbreviations: CA, chronologic age; CNS, central nervous system; NA, not applicable; RT, radiotherapy.

^a^ Include neuroblastoma, germ cell tumor, and Wilms tumor.

### Cross-sectional Annual Change of EA and Comparison of EAA Across CA-Defined Groups

**Figure 1.**
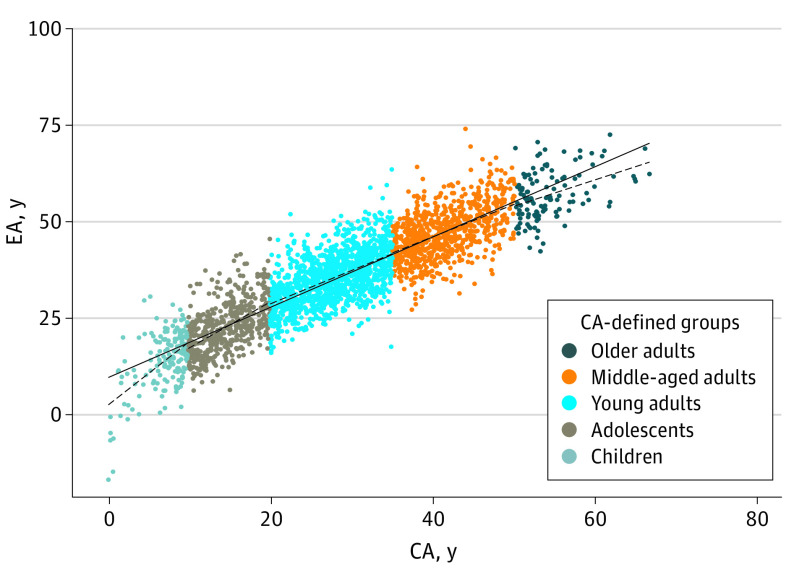
Cross-sectional Trend of Epigenetic Age Using the Levine Clock (EA_Levine) Among Children, Adolescent, and Adult Survivors of Childhood Cancer EA_Levine showed monotonically decreased trend (ie, age slope) across all 5 CA-defined groups (ie, children, adolescents, young adults, middle-aged adults, and older adults). The solid line corresponds to the overall trend across all survivors. CA indicates chronologic age; EA, epigenetic age.

The cross-sectional annual change of EA monotonically decreased across the 5 CA-defined groups (ie, children [1.63], adolescents [1.14], younger adults [0.83], middle-aged adults [0.83], and older adults [0.76]) for EA_Levine (Figure 1; eTable 1 in Supplement 1). When children were used as the reference group, the change rate of EA_Levine was significantly lower in adolescents (β = −0.49; *P* = 6.61 × 10^−3^), younger adults (β = −0.80; *P* = 1.41 × 10^−6^), middle-aged adults (β = −0.80; *P* = 2.01 × 10^−6^), and older adults (β = −0.88; *P* = 3.45 × 10^−5^) (eTable 2 in Supplement 1).

**Figure 2.**
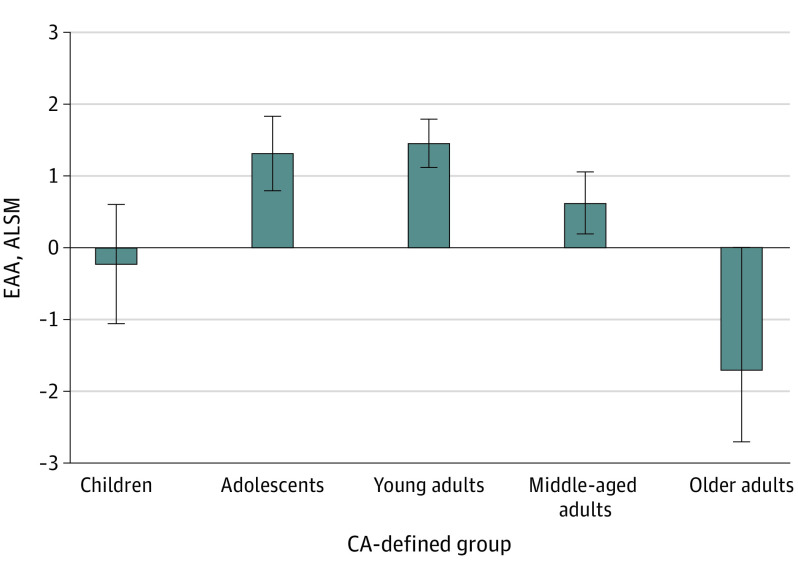
Epigenetic Age Acceleration Using the Levine Clock (Levine EEA) Among Children, Adolescent, and Adult Survivors of Childhood Cancer The distributions of adjusted least square means (ALSMs) of Levine EEA adjusting for sex and treatment exposures in chronologic age (CA)-defined age groups showed negative values for children and older adult survivors compared with positive values for survivors in the other 3 CA-defined groups (adolescents, younger adults, and middle-aged adults). Error bars indicate 95% CI.

The distributions of ALSM of EAA adjusting for sex and treatment exposures in CA-defined age groups showed negative ALSM of Levine EEA for children (−0.22) and older adults (−1.70), whereas participants in the other 3 CA-defined groups had positive ALSM of Levine EEA (adolescents, 1.32; younger adults, 1.46; and middle-aged adults, 0.63) (Figure 2; eTable 3 in Supplement 1).

### Association With Early-Onset Obesity, Severity/Burden of All CHCs, and Late Mortality

Among 690 participants who had EAA measured before age 20 years, 530 had a clinical assessment for obesity. Of these, 219 developed early-onset obesity with CTCAE grade 2 or higher, and 311 did not. An association with obesity was observed for Levine EEA, with a 46% increased risk for each SD increase of EAA (odds ratio [OR], 1.46, 95% CI, 1.19-1.78; *P* = 2.19 × 10^−4^) (Table 2; eTable 4 in Supplement 1).

**Table 2.** Associations Between Levine EEA and Various Outcomes

Outcome and group	OR or HR (95% CI)^a^	*P* value
Early-onset obesity		
Age <20 y	1.46 (1.19-1.78)	2.19 × 10^−4^
Severity burden of all CHCs		
Overall	1.13 (1.03-1.24)	8.03 × 10^−3^
Aged <20 y	1.06 (0.88-1.28)	5.16 × 10^−1^
Aged ≥20 y	1.21 (1.08-1.35)	7.24 × 10^−4^
Late mortality		
Overall	1.75 (1.35-2.26)	1.90 × 10^−5^

Abbreviations: CHCs, chronic health conditions; Levine EEA, epigenetic age acceleration using the Levine clock; OR, odds ratio; HR, hazard ratio.

^a^ Cox proportional hazards regression was used for late mortality, with HR reported; logistic regression was used for other outcomes, with OR reported.

Among 2310 survivors who had at least 1 clinical follow-up assessment, a total of 1652 had high severity/burden scores of CHCs and 658 had low severity/burden scores of CHCs. An association with the dichotomous severity/burden of CHCs (high vs low) was observed for Levine EEA showing a 13% increased risk per SD increase of EAA (OR, 1.13; 95% CI, 1.03-1.24; *P* = 8.03 × 10^−3^) (Table 2). In addition, treatment exposures, including brain RT (OR, 1.91; 95% CI, 1.52-2.41; *P* = 4.40 × 10^−8^) and alkylating agents (OR, 1.32; 95% CI, 1.08-1.61; *P* = 7.15 × 10^−3^), were associated with an increased risk for CHCs, whereas anthracyclines (OR, 0.67; 95% CI, 0.54-0.84; *P* = 3.91 × 10^−4^) were associated with a decreased risk (ie, protective effect) for severity/burden of CHCs (eTable 5 in Supplement 1). When participants who had EAA measured after age 20 years (ie, 3 adult groups) were considered, 1539 had a high severity/burden of CHCs and 597 had a low severity/burden of CHCs. The Levine EEA was associated with severity/burden of CHCs (OR, 1.21; 95% CI, 1.08-1.35; *P* = 7.24 × 10^−4^) (Table 2). When participants who had EAA measured before age 20 years (children and adolescent groups) were considered, 289 had a high severity/burden of CHCs and 313 had a low severity burden of CHCs, with no association between Levine EEA and CHCs.

Among 2522 participants who had mortality data, 70 death events (2.8%) occurred. Time-to-event analysis started from age at DNA sampling for EAA measurement until the death event or date of last contact, whichever occurred earlier. Associations with all-cause mortality were observed for Levine EEA (hazard ratio [HR], 1.75; 95% CI, 1.35-2.26; *P* = 1.90 × 10^−5^) (Table 2). In addition, females had a lower risk of death than males (HR, 0.55; 95% CI, 0.33-0.93; *P* = 2.41 × 10^−2^). Individuals exposed to anthracyclines had a 50% reduction in mortality risk (HR, 0.52; 95% CI, 0.30-0.90; *P* = 1.97 × 10^−2^) (eTable 6 in Supplement 1).

### Comparative Analyses of EAA-Horvath, EAA-Hannum, and EAA-GrimAge

Similar to EA_Levine, the cross-sectional annual change of EA monotonically decreased across the 5 CA-defined groups for EA_Horvath (children, 2.18; adolescents, 0.80; younger adults, 0.66; middle-aged adults, 0.58; and older adults, 0.53). In contrast, the change rates for EA_Hannum (children, 0.52; adolescents, 0.48; younger adults, 0.76; middle-aged adults, 0.66; and older adults, 0.74) and EA_GrimAge (children, 0.59; adolescents, 0.79; younger adults, 0.80; middle-aged adults, 0.80; and older adults 0.62) were highest among younger adults (eFigure 1, eTable 1 in Supplement 1).

Delta age (ie, EA – CA) was also used as the measurement of EAA.^17^ eFigure 2 in Supplement 1 illustrates the difference between EA and CA vs the means of EA and CA in the Bland-Altman plot. There is a clear pattern of steady decrease for delta age across the mean of EA and CA, with the smallest delta ages for individuals with the oldest means of EA and CA for 3 epigenetic clocks. EA_Levine was an exception, with delta age comparable across the means of EA and CA. EA_Horvath showed relatively smaller delta age (ie, an underestimate of EAA) for participants who were children compared with those who were younger adults.

The ALSM of EAA_Hannum was mostly positive, with the highest value for the children (1.93). EAA_Horvath and EAA_Hannum had similar positive ALSM values, with adolescents (EAA_Horvath, 0.93; EAA_Hannum, 1.12) compared with EAA_GrimAge, which had a slightly negative ALSM values in adolescents (−0.09). It is notable that EAA_GrimAge had the lowest variability (based on 95% CIs) with adolescents and older adults having slightly negative values. EA_Horvath and EA_Hannum showed more variability with older adults (eFigure 3 in Supplement 1).

Similar to Levine EEA, EAA_GrimAge was associated with 42% higher risk for participants to develop early-onset obesity (OR, 1.42; 95% CI, 1.15-1.75; *P* = 1.30 × 10^−3^). In contrast, although still significant, the OR was lower with EAA_Horvath (OR, 1.25; 95% CI, 1.03-1.51; *P* = 2.44 × 10^−2^); EAA_Hannum was not associated with early-onset obesity (eTable 4 in Supplement 1). Similar to Levine EEA, similar effect sizes were noted with EAA_Hannum (OR, 1.14) and EAA_GrimAge (OR, 1.16) for the severity/burden of CHCs, but not EAA_Horvath (eTable 5 in Supplement 1). Also similar to Levine EEA, EAA_GrimAge was associated with late mortality (HR, 1.63; 95% CI, 1.32-2.02; *P* = 5.13 × 10^−6^) (eTable 6 in Supplement 1).

## Discussion

We found that the cross-sectional annual change of EA vs CA and EAA differ across CA groups. We further noted that increased EAA was associated with early-onset obesity among children and adolescents, and EAA was associated with severity/burden of all CHCs, as well as late mortality among all participants.

Children, followed by adolescents, had the highest change rate of EA_Horvath or EA_Levine, which may be reflective of fast growth and puberty. EA_Hannum and EA_GrimAge were different, with younger adults showing the highest change rate across all CA groups. EA_Horvath is the only epigenetic clock that included samples collected from children and adolescents in the model training, so it might be the most reliable clock for evaluating epigenetic age in this age range.^23^ Note that the mean age of individuals donating DNA for the training set was 66 years for the Levine clock, without available documentation of the exact age range. However, all 4 clocks showed lower rates of change of EA in older adults. By comparing EAA among survivors of childhood cancer, EAA_Horvath and Levine EEA were negative for children and older adults, which corroborated systematic underestimation of EA and EAA in older individuals previously reported in the general population.^21^ Our findings suggest that CA groups need to be considered when carrying out studies using EAA as risk factors/predictors to avoid spurious association.

Our results showed an association between EAA and early-onset obesity. There is a 46% higher risk in developing early-onset obesity per SD increase of Levine EEA. Similarly, such risk is 42% higher for EAA_GrimAge. In contrast, although associated with early-onset obesity, the OR with EAA_Horvath was lower, and EAA_Hannum was not associated with early-onset obesity. A temporal association between EAA measured in children and adolescents and early-onset obesity suggests that early intervention with respect to EA may prevent or remediate childhood obesity, which may ultimately ameliorate many other adulthood CHCs.^12,13,14^ Children and adolescents are potentially the best candidates for antiaging interventions among survivors of childhood cancer. Cranial radiation exposure also increased the risk for early-onset obesity, independent of the epigenetic clock metrics. This was consistent in all 4 clocks, with a 2-fold increased risk comparing individuals who received vs did not receive cranial RT. Considering the previous study reporting that cranial RT was not associated with Levine EEA,^11^ EAA and cranial RT appear to be independent risk factors for early-onset obesity. Childhood cancer survivors tend to have less healthy lifestyles, including being sedentary, having lower levels of physical activity, and having unhealthy eating habits.^24,25,26,27,28^ Future work should evaluate whether EAA mediates the association between lifestyles as modifiable risk factors and risk of early-onset obesity, which may provide supporting evidence for lifestyle interventions to slow EAA and prevent early-onset obesity.

Epigenetic age acceleration was also associated with the overall health outcome metric as defined by the severity/burden of all CHCs. The Levine and GrimAge clocks are better designed to reflect late-life disease burden and late mortality using methylation patterns.^29,30,31^ Higher EAA based on these clocks was associated with an increased risk for developing severe disease burden. When CA groups were adjusted for in the model, a better association signal was detected. When stratified by CA-defined groups, the association between Levine EEA and increased risk in severity/burden of CHCs seemed to be significant in adults, but not in children and adolescents (perhaps due to the lower severity/burden of CHCs within children and adolescents and smaller sample sizes). Regarding cancer treatments, cranial RT was associated with severity/burden of CHCs. Moreover, exposure to alkylating agents was associated with an increased risk of developing a severe disease burden, but exposure to anthracyclines appeared to have a protective effect, reducing the risk of developing severe disease burden. This could be explained by the inverse association between cranial RT and anthracyclines, ie, survivors who received anthracyclines were more unlikely exposed to cranial RT. Finally, Levine EEA or EAA_GrimAge was strongly associated with late-mortality among survivors with greater than 50% increased risk per SD increase of EAA.

### Limitations

This study has limitations. First, our study of EAA across CA-defined groups is cross-sectional by design, ie, the trajectory was based on this study population instead of measuring the EA and EAA at multiple time points for the same individual. Second, although we have considered sex, age, and cancer treatments as covariates for adjustment in our models, other disease-specific risk factors, as well as unmeasured confounding factors, were not considered. Third, we focused on survivors of European ancestry, so findings from our study cannot be extrapolated to survivors of African ancestry or other underrepresented racial and ethnic groups without further validation. Fourth, future expanded research in multicenter cohorts (eg, Childhood Cancer Survivor Study)^32^ is warranted to corroborate or refute our findings based on the SJLIFE study.

## Conclusions

This cross-sectional study found a variable rate of change in epigenetic age across chronologic age groups, with most clocks showing an increased rate of change in children and adolescents and a decreased rate of change in older adults. Our findings also suggest that EAA measured in children and adolescent survivors of childhood cancer is associated with early-onset obesity among younger individuals, and the severity/burden of all CHCs as well as late mortality among all survivors. Their young chronologic age at presentation highlights the importance of potential early entry point for antiaging interventions including nonpharmacologic (eg, lifestyle modifications) and pharmacologic (eg, DNA methylation or demethylating agents) strategies to reduce morbidity and mortality during survivorship care.

## Notes

Supplement 1. **eFigure 1.** Cross-sectional Trend of Epigenetic Age Among Children and Adolescents, and Adult Survivors of Childhood Cancer **eFigure 2.** Mean-Difference (Bland-Altman) Plots Showing the Difference Between Epigenetic Age (Four Different Clocks) and Chronological Age **eFigure 3.** Epigenetic Age Acceleration Differs by CA-Defined Groups **eTable 1.** Cross-sectional Annual Change Rate of Epigenetic Age for Survivors Within Each Chronological Age-Defined Group **eTable 2.** Multivariable Linear Regression of EA_Levine Against Age at DNA Sampling (Chronological Age) **eTable 3.** Distributions of ALSM of EAA Adjusting for Sex and Treatment Exposures in Each Chronological Age-Defined Group **eTable 4.** Associations Between EAA and Early-Onset Obesity **eTable 5.** Associations Between EAA and Severity/Burden of All CHCs **eTable 6.** Associations Between EAA and Late-Mortality

Supplement 2.
Data Sharing Statement
