# Supplementary material for: Epigenetic Age in Peripheral Blood Among Children, Adolescent, and Adult Survivors of Childhood Cancer
Source: JAMA Netw Open. 2023 Apr 28;6(4):e2310325. doi: 10.1001/jamanetworkopen.2023.10325 (PMC10148192; doi:10.1001/jamanetworkopen.2023.10325)
Supplement: Supplement 1. — eFigure 1. Cross-sectional Trend of Epigenetic Age Among Children and Adolescents, and Adult Survivors of Childhood Cancer eFigure 2. Mean-Difference (Bland-Altman) Plots Showing the Difference Between Epigenetic Age (Four Different Clocks) and Chronological Age eFigure 3. Epigenetic Age Acceleration Differs by CA-Defined Groups eTable 1. Cross-sectional Annual Change Rate of Epigenetic Age for Survivors Within Each Chronological Age-Defined Group eTable 2. Multivariable Linear Regression of EA_Levine Against Age at DNA Sampling (Chronological Age) eTable 3. Distributions of ALSM of EAA Adjusting for Sex and Treatment Exposures in Each Chronological Age-Defined Group eTable 4. Associations Between EAA and Early-Onset Obesity eTable 5. Associations Between EAA and Severity/Burden of All CHCs eTable 6. Associations Between EAA and Late-Mortality [file jamanetwopen-e2310325-s001.pdf]

Supplementary Online Content

Plonski NM, Chen C, Dong Q, et al. Epigenetic age in peripheral blood among children, adolescent, and adult survivors of childhood cancer. *JAMA Netw Open*. 2023;6(4):e2310325. doi:10.1001/jamanetworkopen.2023.10325

eFigure 1. Cross-sectional Trend of Epigenetic Age Among Children and Adolescents, and Adult Survivors of Childhood Cancer

eFigure 2. Mean-Difference (Bland-Altman) Plots Showing the Difference Between Epigenetic Age (Four Different Clocks) and Chronological Age

eFigure 3. Epigenetic Age Acceleration Differs by CA-Defined Groups

eTable 1. Cross-sectional Annual Change Rate of Epigenetic Age for Survivors Within Each Chronological Age-Defined Group

eTable 2. Multivariable Linear Regression of EA_Levine Against Age at DNA Sampling (Chronological Age)

eTable 3. Distributions of ALSM of EAA Adjusting for Sex and Treatment Exposures in Each Chronological Age-Defined Group

eTable 4. Associations Between EAA and Early-Onset Obesity

eTable 5. Associations Between EAA and Severity/Burden of All CHCs

eTable 6. Associations Between EAA and Late-Mortality

This supplementary material has been provided by the authors to give readers additional information about their work.

© 2023 Plonski NM et al. *JAMA Network Open.*

eFigure 1. Cross-sectional trend of epigenetic age among children and adolescents, and adult survivors of childhood cancer.

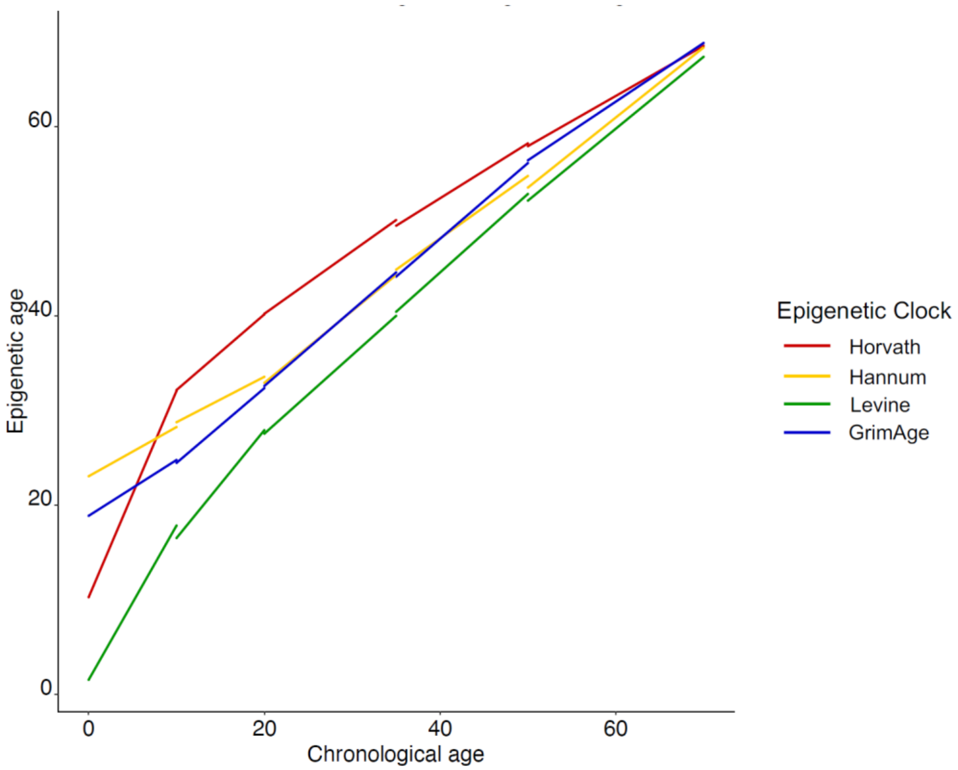

Epigenetic aging among children, adolescents, young adults, adults and older adults shows significantly different trend. EA_Horvath shown in red and EA_Levine shown in green had decreased trend (i.e., age slopes) across age groups from children to old adults. EA_Hannum shown in yellow and EA_GrimAge shown in blue had the highest trend in young adults and lower trend in children or old adults.

© 2023 Plonski NM et al. *JAMA Network Open.*

eFigure 2. Mean-difference (Bland-Altman) plots showing the difference between epigenetic age (four different clocks) and chronological age.

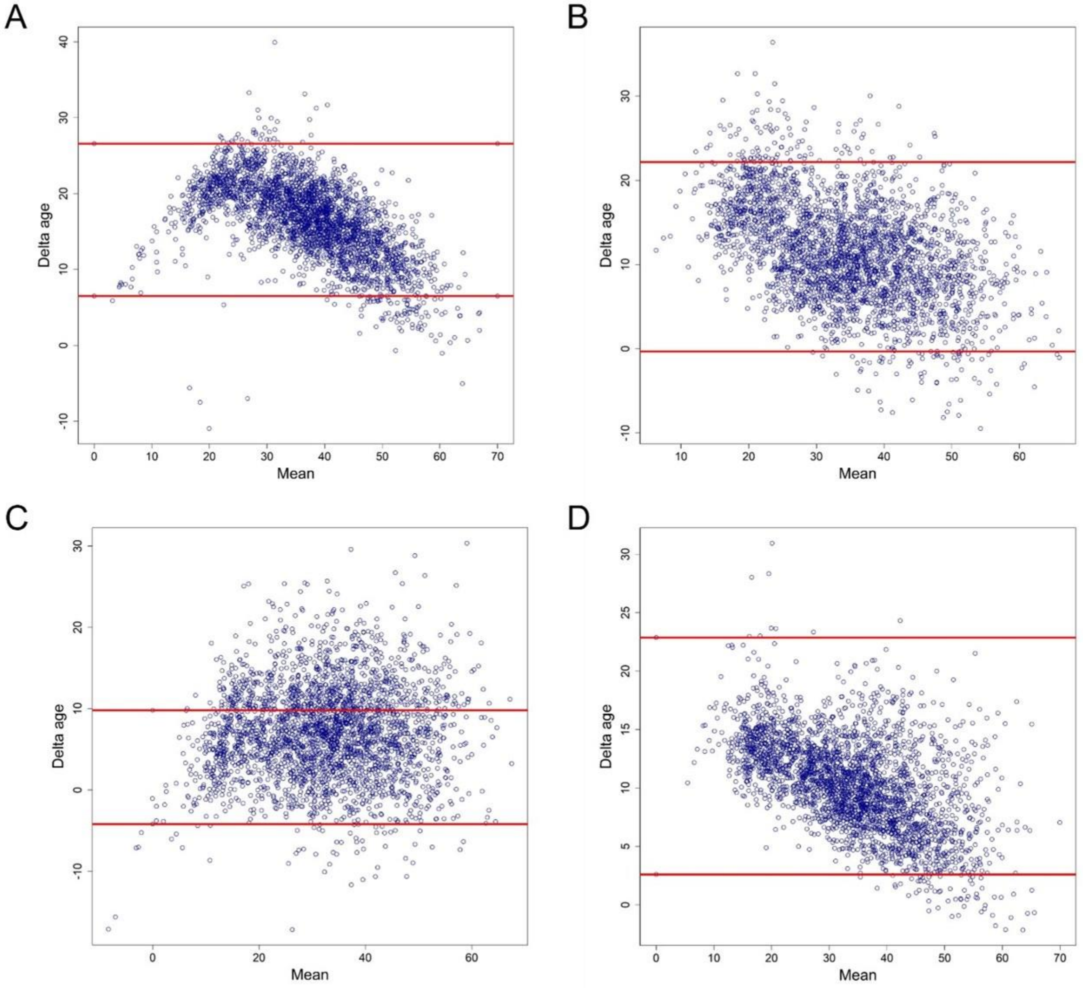

Delta age as calculated by CA-EA is shown in these Bland Altman plots. (A) We confirm EA_Horvath clock has smaller delta age with children, adolescents, and older adults. (B) EA_Hannum clock confirmed older adults have lower delta age but not children. (C) EA_Hannum had more variability but still had a negative delta age for children and older adults. (D) EA_GrimAge agrees with EA_Hannum with a steady decrease in delta age as getting older.

© 2023 Plonski NM et al. *JAMA Network Open.*

eFigure 3. Epigenetic age acceleration differs by CA-defined groups.

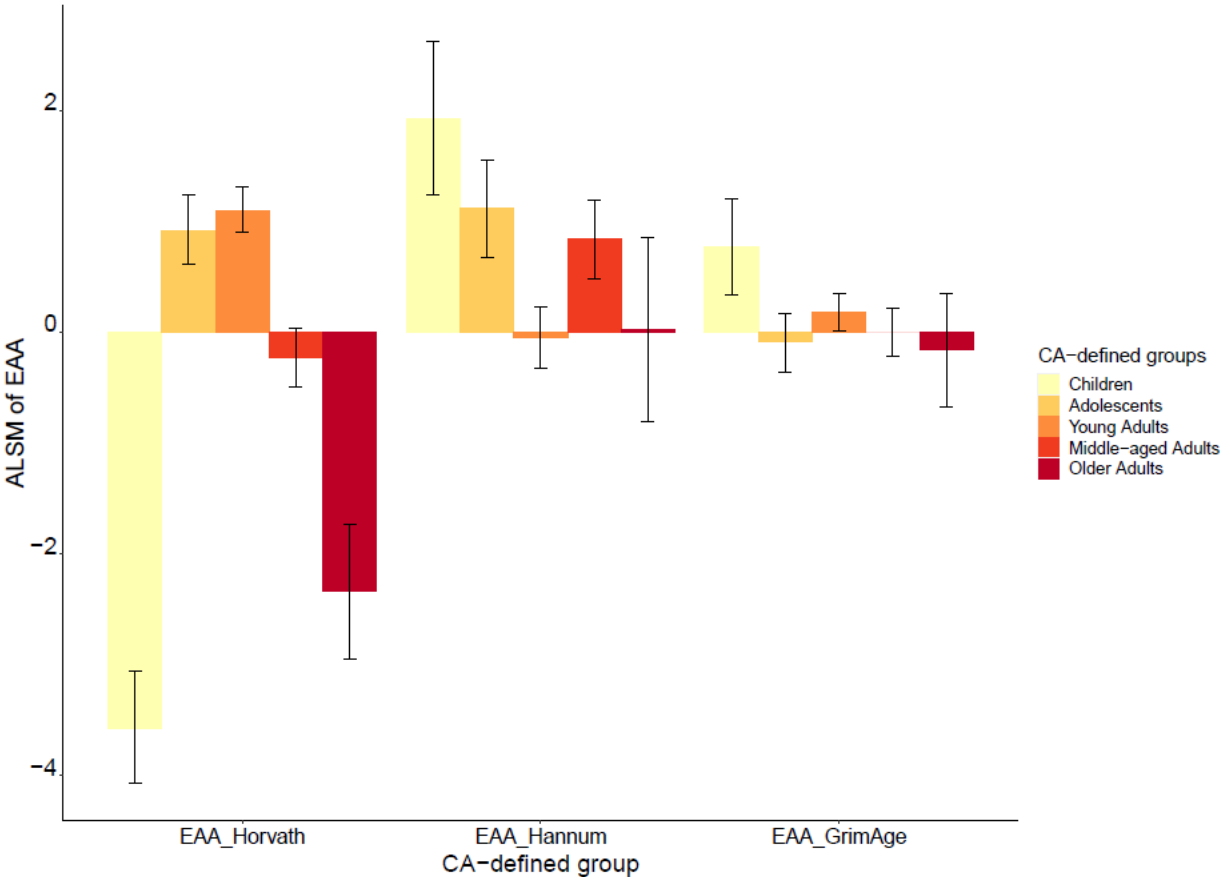

EAA_Horvath showed lower negative ALSM for children, middle-aged adults and old adults, and higher positive ALSM for adolescents and young adults; EAA_Hannum showed a positive ALSM for all CA groups except of young adults; EAA_GrimAge had negative ALSM for both adolescents, middle-aged adults and old adults but positive ALSM for children and young adults. ALSM, adjusted least square means; CA, chronological age; EAA, epigenetic age acceleration.

© 2023 Plonski NM et al. *JAMA Network Open.*

eTable 1. Cross-sectional annual change rate of epigenetic age for survivors within each chronological age-defined group.

	Coefficients	EA_Horvath	EA_Hannum	EA_Levine	EA_GrimAge
Children	Intercept	10.26	23.04	1.53	18.86
	Beta	2.18	0.52	1.63	0.59
Adolescents	Intercept	24.15	23.95	5.11	16.55
	Beta	0.8	0.48	1.14	0.79
Young adults	Intercept	27.01	17.71	10.94	16.58
	Beta	0.66	0.76	0.83	0.8
Middle-aged adults	Intercept	29.21	21.77	11.37	16.13
	Beta	0.58	0.66	0.83	0.8
Old adults	Intercept	31.42	16.54	14.16	25.42
	Beta	0.53	0.74	0.76	0.62

© 2023 Plonski NM et al. *JAMA Network Open.*

eTable 2. Multivariable linear regression of EA_Levine against age at DNA sampling (chronological age).

Coefficient	Beta	SE	*P*
(Intercept)	1.53	1.27	2.26E-01
Sample Age	1.63	0.16	<2.00E-16
Adolescents	3.58	1.75	4.09E-02
Young adults	9.40	1.60	4.62E-09
Middle-aged adults	9.84	2.35	3.03E-05
Old adults	12.62	7.47	9.12E-02
Gender	-0.99	0.20	9.81E-07
Chest-RT	3.36	0.26	<2.00E-16
Alkylating agent, classic	1.70	0.21	1.62E-15
Epipodophyllotoxins	0.89	0.23	8.80E-05
Sample Age:Adolescents	-0.49	0.18	6.61E-03
Sample Age:Young adults	-0.80	0.17	1.41E-06
Sample Age:Middle-aged adults	-0.80	0.17	2.01E-06
Sample Age:Old adults	-0.88	0.21	3.45E-05

Abbreviations: SE, standard error.

© 2023 Plonski NM et al. *JAMA Network Open.*

eTable 3. Distributions of ALSM of EAA adjusting for sex and treatment exposures in each chronological age-defined group.

Age Group	EAA_Horvath			EAA_Hannum			EAA_Levine			EAA_GrimAge		
	ALSM	95% CI lower bound	95% CI upper bound	ALSM	95% CI lower bound	95% CI upper bound	ALSM	95% CI lower bound	95% CI upper bound	ALSM	95% CI lower bound	95% CI upper bound
Children	-3.57	-4.07	-3.06	1.93	1.24	2.62	-0.22	-1.05	0.61	0.77	0.34	1.20
Adolescents	0.92	0.61	1.24	1.12	0.68	1.55	1.32	0.81	1.84	-0.09	-0.36	0.17
Young Adults	1.10	0.90	1.31	-0.05	-0.33	0.23	1.46	1.13	1.80	0.18	0.01	0.35
Middle-aged Adults	-0.23	-0.49	0.04	0.84	0.48	1.19	0.63	0.20	1.06	0.00	-0.22	0.22
Older Adults	-2.34	-2.95	-1.73	0.03	-0.81	0.86	-1.70	-2.70	-0.70	-0.16	-0.68	0.35

Abbreviations: ALSM, Adjusted Least Square Means; EAA, Epigenetic Accelerated Age; CI, Confidence interval.

© 2023 Plonski NM et al. *JAMA Network Open.*

eTable 4. Associations between EAA and early-onset obesity.

Coefficients	EAA_Horvath			EAA_Hannum			EAA_Levine			GrimAge		
	OR	95% CI	*P*	OR	95% CI	*P*	OR	95% CI	*P*	OR	95% CI	*P*
(Intercept)	0.73	0.52-1.02	6.64E-02	0.72	0.51-1.01	5.69E-02	0.81	0.57-1.15	2.34E-01	0.72	0.51-1.01	5.53E-02
EAA	1.25	1.03-1.51	2.44E-02	1.18	0.99-1.41	6.85E-02	1.46	1.19-1.78	**2.19E-04**	1.42	1.15-1.75	**1.30E-03**
Gender	0.88	0.62-1.26	4.97E-01	0.84	0.59-1.20	3.52E-01	0.89	0.62-1.27	5.21E-01	1.07	0.72-1.57	7.46E-01
Brain-RT	1.77	0.94-3.34	7.64E-02	2.03	1.08-3.82	2.88E-02	1.81	0.96-3.41	6.76E-02	1.91	1.01-3.60	4.52E-02
Neck-RT	0.67	0.21-2.14	4.99E-01	0.71	0.22-2.25	5.55E-01	0.73	0.23-2.36	6.02E-01	0.75	0.23-2.44	6.34E-01
Chest-RT	0.58	0.21-1.58	2.83E-01	0.62	0.23-1.68	3.47E-01	0.49	0.18-1.37	1.74E-01	0.63	0.23-1.75	3.73E-01
Abdomen-RT	0.81	0.28-2.31	6.90E-01	0.87	0.30-2.48	7.94E-01	0.91	0.32-2.62	8.61E-01	0.90	0.31-2.59	8.44E-01
Pelvis-RT	1.26	0.39-4.01	6.98E-01	1.13	0.36-3.58	8.36E-01	1.12	0.35-3.61	8.46E-01	0.91	0.28-2.96	8.74E-01
Alkylating agent, classic	0.81	0.51-1.28	3.59E-01	0.79	0.50-1.26	3.28E-01	0.74	0.47-1.18	2.13E-01	0.75	0.47-1.19	2.19E-01
Epipodophyllotoxins	0.92	0.61-1.39	6.95E-01	0.91	0.61-1.37	6.66E-01	0.86	0.57-1.30	4.79E-01	0.84	0.56-1.28	4.22E-01
Anthracyclines	1.34	0.82-2.20	2.48E-01	1.39	0.85-2.29	1.90E-01	1.21	0.73-2.00	4.62E-01	1.29	0.78-2.13	3.22E-01

Abbreviations: OR, odds ratio; EAA, Epigenetic Accelerated Age; CI, Confidence interval.

© 2023 Plonski NM et al. *JAMA Network Open.*

eTable 5. Associations between EAA and severity/burden of all CHCs.

Coefficients	EAA_Horvath			EAA_Hannum			EAA_Levine			EAA_GrimAge		
	OR	95% CI	*P*	OR	95% CI	*P*	OR	95% CI	P	OR	95% CI	*P*
(Intercept)	1.20	0.83-1.76	3.34E-01	1.11	0.77-1.60	5.84E-01	1.22	0.85-1.77	2.84E-01	1.11	0.77-1.60	5.70E-01
EAA	1.04	0.95-1.14	3.76E-01	1.14	1.04-1.25	**3.63E-03**	1.13	1.03-1.24	**8.03E-03**	1.16	1.05-1.28	**2.91E-03**
Attained Age	0.99	0.98-1.00	4.66E-02	0.99	0.98-1.00	5.75E-02	0.99	0.98-1.00	5.60E-02	0.99	0.98-1.00	3.93E-02
Gender	1.10	0.93-1.31	2.61E-01	1.13	0.95-1.34	1.81E-01	1.12	0.94-1.33	2.00E-01	1.21	1.01-1.46	4.04E-02
Adolescents	0.70	0.47-1.03	7.15E-02	0.75	0.51-1.10	1.36E-01	0.71	0.48-1.04	7.56E-02	0.76	0.52-1.11	1.61E-01
Young adults	1.54	1.05-2.27	2.78E-02	1.72	1.19-2.49	4.12E-03	1.57	1.08-2.27	1.69E-02	1.68	1.16-2.42	6.01E-03
Middle-aged adults	4.41	2.77-7.02	4.11E-10	4.70	2.97-7.42	3.58E-11	4.47	2.83-7.06	1.44E-10	4.78	3.02-7.56	2.42E-11
Old adults	7.04	3.23-15.35	9.50E-07	7.47	3.42-16.30	4.40E-07	7.31	3.35-15.94	5.84E-07	7.58	3.47-16.58	3.92E-07
Brain-RT	1.88	1.49-2.37	8.79E-08	1.91	1.52-2.41	4.40E-08	1.91	1.52-2.41	**4.40E-08**	1.93	1.53-2.43	3.05E-08
Neck-RT	1.05	0.70-1.57	8.20E-01	1.02	0.68-1.54	9.17E-01	1.02	0.68-1.54	9.08E-01	1.05	0.70-1.58	8.11E-01
Chest-RT	1.10	0.73-1.66	6.54E-01	1.08	0.72-1.64	6.99E-01	1.07	0.71-1.62	7.42E-01	1.09	0.72-1.65	6.72E-01
Abdomen-RT	1.06	0.70-1.61	7.72E-01	1.05	0.69-1.59	8.26E-01	1.04	0.68-1.58	8.56E-01	1.06	0.70-1.61	7.76E-01
Pelvis-RT	1.51	1.00-2.29	5.17E-02	1.51	0.99-2.29	5.33E-02	1.50	0.99-2.28	5.60E-02	1.48	0.98-2.25	6.20E-02
Alkylating agent, classic	1.36	1.11-1.66	2.97E-03	1.35	1.10-1.64	3.70E-03	1.32	1.08-1.61	**7.15E-03**	1.33	1.09-1.62	5.55E-03
Epipodophyllotoxins	0.86	0.71-1.05	1.33E-01	0.85	0.70-1.04	1.13E-01	0.84	0.69-1.02	8.54E-02	0.84	0.69-1.03	8.75E-02
Anthracyclines	0.68	0.55-0.85	6.20E-04	0.69	0.55-0.85	6.81E-04	0.67	0.54-0.84	**3.91E-04**	0.67	0.54-0.83	2.88E-04

Abbreviations: OR, odds ratio; EAA, Epigenetic Accelerated Age; CI, Confidence interval.

© 2023 Plonski NM et al. *JAMA Network Open.*

eTable 6. Associations between EAA and late-mortality.

Coefficients	Horvath			Hannum			Levine			GrimAge		
	OR	95%CI	*P*	OR	95%CI	*P*	OR	95%CI	*P*	OR	95%CI	*P*
Gender	0.53	0.32-0.88	1.48E-02	0.54	0.33-0.91	1.93E-02	0.55	0.33-0.93	2.41E-02	0.77	0.45-1.31	3.30E-01
EAA	1.11	0.83-1.48	4.69E-01	1.13	0.89-1.45	3.16E-01	1.75	1.35-2.26	**1.90E-05**	1.63	1.32-2.02	**5.13E-06**
Sample Age	1.08	1.05-1.11	6.43E-07	1.07	1.04-1.10	6.07E-07	1.08	1.05-1.11	3.61E-08	1.07	1.04-1.10	1.20E-06
Brain-RT	0.96	0.55-1.66	8.75E-01	0.99	0.56-1.72	9.59E-01	1.15	0.66-2.00	6.25E-01	1.09	0.62-1.90	7.67E-01
Neck-RT	1.97	0.81-4.79	1.33E-01	1.92	0.79-4.70	1.51E-01	1.70	0.69-4.20	2.48E-01	1.65	0.69-3.97	2.61E-01
Chest-RT	1.88	0.70-5.06	2.10E-01	1.87	0.69-5.04	2.18E-01	1.76	0.65-4.80	2.68E-01	2.18	0.82-5.76	1.16E-01
Abdomen-RT	1.57	0.65-3.75	3.13E-01	1.58	0.66-3.77	3.08E-01	1.23	0.51-2.96	6.39E-01	1.50	0.62-3.64	3.66E-01
Pelvis-RT	0.91	0.42-1.98	8.20E-01	0.92	0.42-2.01	8.37E-01	1.03	0.48-2.20	9.41E-01	0.93	0.43-2.03	8.57E-01
Alkylating agent, classic	1.35	0.77-2.36	2.97E-01	1.34	0.77-2.35	3.05E-01	1.13	0.64-1.98	6.80E-01	1.21	0.69-2.14	5.05E-01
Epipodophyllotoxins	1.06	0.52-2.20	8.66E-01	1.07	0.52-2.22	8.54E-01	0.99	0.48-2.04	9.74E-01	1.00	0.48-2.09	9.90E-01
Anthracyclines	0.49	0.28-0.85	1.16E-02	0.50	0.29-0.86	1.32E-02	0.52	0.30-0.90	1.97E-02	0.47	0.27-0.83	8.53E-03

Abbreviations: OR, odds ratio; EAA, Epigenetic Accelerated Age; CI, Confidence interval.

© 2023 Plonski NM et al. *JAMA Network Open.*
