# Supplementary material for: Epigenetic Age in Peripheral Blood Among Children, Adolescent, and Adult Survivors of Childhood Cancer
Source: JAMA Netw Open. 2023 Apr 28;6(4):e2310325. doi: 10.1001/jamanetworkopen.2023.10325 (PMC10148192; doi:10.1001/jamanetworkopen.2023.10325)
Supplement: Supplement 2. — Data Sharing Statement [file jamanetwopen-e2310325-s002.pdf]

Data Sharing Statement

Plonski. Epigenetic Age in Peripheral Blood Among Children, Adolescent, and Adult Survivors of Childhood Cancer. *JAMA Netw Open*. Published April 28, 2023. doi:10.1001/jamanetworkopen.2023.10325

Data
Data available: Yes
Data types: Deidentified participant data
How to access data: The data is accessible through the St. Jude Cloud (https://stjude.cloud)
When available: With publication

Supporting Documents
Document types: None

Additional Information
Who can access the data: researchers whose proposed use of the data has been approved
Types of analyses: for research purpose
Mechanisms of data availability: after approval of a proposal by the data access committee and with a signed data access agreement
